# Experiences of internet-based cognitive behavioural therapy for depression and anxiety among Arabic-speaking individuals in Sweden: a qualitative study

**DOI:** 10.1186/s12888-021-03297-w

**Published:** 2021-06-03

**Authors:** Tomas Lindegaard, Fatima Kashoush, Sara Holm, Asala Halaj, Matilda Berg, Gerhard Andersson

**Affiliations:** 1 Department of Behavioural Sciences and Learning, Linköping University, SE-581 83 Linköping, Sweden; 2 Department of Psychology, The Hebrew University of Jerusalem, Jerusalem, Israel; 3 Department of Clinical Neuroscience, Karolinska Institute, Stockholm, Sweden; 4 Department of Biomedical and Clinical Sciences, Linköping University, Linköping, Sweden

**Keywords:** Depression, Thematic analysis, Anxiety, Internet-based cognitive behaviour therapy, Culturally adapted psychotherapy, Qualitative, Transcultural, Refugees

## Abstract

**Background:**

Internet-delivered cognitive behavioural therapy (ICBT) is a promising treatment for refugee and immigrant populations suffering from common mental disorders. The aim of the present study was to investigate experiences of participating in a guided ICBT program among resettled Arabic-speaking individuals suffering from symptoms of anxiety and depression.

**Methods:**

Ten individuals who had previously received ICBT consented to participate and were interviewed using semi-structured telephone interviews. The interviews were conducted 10 months after treatment termination. Data were transcribed and analysed using a Thematic Analysis framework.

**Results:**

The Thematic Analysis resulted in five overarching themes 1) *The importance of being seen,* 2) *New ways of knowing and doing,* 3) *Treatment format not for everyone,* 4) *Changing attitudes towards mental health and help-seeking* and 5) *The healthcare system as a complex puzzle.* Participants described varying levels of success in applying the new information learned from the treatment in their everyday lives. The results also indicate that participation in the ICBT program to some extent mitigated mental health stigma and acted as a precursor to other forms of treatment seeking.

**Conclusions:**

The findings in the present study are largely in line with previous qualitative research studies on ICBT participants. Future research should investigate whether a more explicit focus on refugee-specific stressors and barriers to treatment engagement and implementation can increase adherence to ICBT programs in this population.

**Supplementary Information:**

The online version contains supplementary material available at 10.1186/s12888-021-03297-w.

## Background

According to the UNHCR, around 80 million people worldwide are currently forcibly displaced, with many originating from the Arab region [1]. Although a majority are hosted by developing countries, a substantial minority have also applied for asylum in the European countries, for example in Sweden and Germany. Previous research has established that forcibly displaced people suffer from elevated levels of common mental disorders such as depression, anxiety and post-traumatic stress [2–5]. The mental health problems experienced by this group have been shown to be related to pre-migration exposure to armed conflict, persecution and violence, as well as post-migration experiences including economic adversity, discrimination and uncertainty regarding if one will be granted an asylum [6–9].

However, despite the high prevalence of mental disorders, research has shown that both refugees and other migrants underutilize mental health services compared to the general population [10–12]. Barriers against seeking and accessing help include structural factors such as language barriers, financial and logistic issues as well as lack of knowledge of how the health care system works. Other barriers involve socio-cultural factors such as stigmatization of mental health problems, privacy concerns and perceptions that caretakers will not understand their problems due to cultural or linguistic differences [10, 11, 13–16]. These barriers can also include religious reasons or supernatural explanatory models for mental health problems, such as *djinn* possession, which can predispose individuals to engage in traditional healing practices rather than seek help through the formal healthcare system [17–19].

In light of the barriers against accessing mental health services mentioned above, some researchers have suggested that Internet-delivered psychological interventions could serve as a way to increase access and uptake of mental health services [15, 20]. Previous research underscores the importance of culturally adapting psychological interventions to make them more aligned with the norms, values and socioeconomic context of the target group [21–23]. Such adaptions have been found to increase treatment effects [24]. A number of randomized controlled trials (RCTs) of culturally adapted Internet-delivered cognitive behavior therapy (ICBT) targeting various populations have now been published with promising results [25–29]. These studies indicate that culturally adapted ICBT has moderate to large effects on symptoms of common mental health disorders compared to inactive control conditions. However, in three of these studies there was also a substantial drop-out rate of around 40% [26, 28, 29], which is higher than what is usually seen in ICBT treatments [30]. The reasons for this high dropout rate are not well understood, although it has been hypothesized that various post-migratory stressors (such as economic adversity, lack of social support and the stress inherent in the asylum-seeking process) could contribute, as well as insufficient cultural adaptation which could make the treatment less relevant to participants [28].

One way to get a more in-depth understanding of participants’ experiences of ICBT programs, including factors that contribute to as well as hinder engagement and adherence, is to use qualitative methods. There are, to our knowledge, no previous qualitative studies investigating the experiences of participants in culturally adapted ICBT studies. However, a number of qualitative studies have been published on participants in ICBT studies aimed at Western populations. These studies have investigated participants’ experiences of the treatment and perception of factors that contribute to treatment adherence and effectiveness [31–39].

A recent meta-synthesis of the acceptability and usability of digital health interventions (DHI) for adults with anxiety, depression and somatoform disorders found three overarching themes across studies, named *initial motivations and approaches to DHIs, personalization of treatment* and *the value of receiving personal support in DHIs* [40]. The first theme included two sub-themes which pointed to the importance of initial motivations and approach to treatment in relation to the perceived usefulness of the DHIs. Participants who approached the intervention with a sense of hope and actively applied the treatment principles seemed to benefit more from treatment than those who adopted a more passive approach. The second theme and related sub-themes described how the flexible format associated with DHIs had different effects for different participants. While some participants appreciated the accessibility and flexibility associated with the DHIs, for others the lack of structure and accountability led to disengagement with the DHI. The anonymity of the internet format was also perceived differently. For most participants this helped them trust and engage with the DHI, however, some participants also expressed concerns about data security. The final theme, which included four sub-themes, described how responsive human support was important for motivation and therapeutic engagement with the DHI and that some participants expressed a wish for more human contact and support [40].

Other important themes, outside the scope of the above mentioned meta-synthesis, found in previous qualitative research include increased self-efficacy [31, 35, 36] as well as increased self-acceptance and understanding/awareness of problems [31, 33, 35, 36] following ICBT. Some participants also mention other negative effects of the treatment format such as the treatment being too time-consuming or feeling pressured by the tight time-schedule [36, 37], lack of tailoring of the treatment to their specific needs [33, 36], as well as increased feelings of shame or hopelessness due to lack of treatment effects [31, 36, 37]. After treatment completion, some participants are able to recall specific treatment techniques [34, 39], although participants also differ in the extent that they actively make use of treatment strategies [39]. Finally, some participants also mention that the treatment acted as a precursor for other treatment seeking, making it easier for them to take the step to seek additional care [33].

To summarize, while culturally adapted ICBT shows promise as a treatment for displaced persons re-settled in Western countries, little is known about participants’ experiences of the programs or the reasons behind the high attrition rates found in some studies. Qualitative research on Western participants in ICBT studies suggest several important themes related to how these programs could be made more effective, however, little is known in regard to what extent these findings transfer to non-western populations. The aim of the present study was to investigate the experience of undergoing a culturally adapted ICBT trial among Arabic-speaking immigrants and refugees residing in Sweden. The study was conducted alongside a randomized controlled trial (RCT) which investigated the efficacy of culturally adapted ICBT compared to a wait-list control condition [28].

## Method

### Study design

As mentioned above, the study was a qualitative follow-up of a previous randomized controlled trial and was approved by the Regional Board of Ethics of Linköping, Sweden, number 2017/488–31 [28]. The original trial was preregistered at clinicaltrials.gov, ID number NCT03496350. Participants were assigned to either 8-week ICBT treatment or a wait-list control condition. All participants in the wait-list received the same treatment after the 8-week waiting period and had thus received the same treatment by the time of the follow-up assessment [28]. Originally, the aim of the present study also included a quantitative follow-up of study participants 10 months after treatment termination, however due to a low response rate, we chose to only present descriptive statistics for participants with regards to the quantitative outcome measures.

### Participants and recruitment

The participants consisted of a sub-set of participants from the original RCT. In the original RCT, participants were recruited through advertisements in social media as well as through advertisements in clinics targeting immigrant and refugee health. All interested participants had to complete an online informed consent form as well as a number of screening questionnaires. After the screening was completed, participants were telephoned for a short clinical interview before starting treatment. Inclusion criteria included being able to read and write Arabic, having elevated symptoms of depression and/or anxiety as measured on the Patient Health Questionnaire-9 (PHQ-9) [41] and the ﻿Generalized Anxiety Disorder-7 (GAD-7) [42], currently reside in Sweden and be above 18 years of age. Exclusion criteria included having a severe mental illness such as bipolar disorder or schizophrenia, suicidal ideation, substance or alcohol abuse or other ongoing psychological treatment. For more details on the recruitment process for the original trial, see [28]. There was no difference with regards to any of the pre-treatment or sociodemographic variables between those who completed the post-treatment assessment and those who did not [28].

At the 10-month follow-up, all participants received an email asking if they were willing to participate in a follow-up interview regarding their experience of the treatment. The email included a link to a separate page with the quantitative outcome assessment, which included the measures listed under the heading quantitative measures below. This page also included an online informed consent form for participating in the qualitative interviews. Participants were also able to choose in which language they wanted to conduct the interview and schedule a time for the phone call with an interviewer. The outcome assessment was completed before participating in the qualitative interviews. Of the 59 participants included in the original trial, 17 completed the 10-month follow-up assessment, and 10 consented to being interviewed for the qualitative follow-up, i.e. a convenience sample. Of those 10, all but one had completed the post-treatment assessment in the original trial. Descriptive statistics of study participants can be found in Table 1.

**Table 1:** Sociodemographic characteristics of participants at baseline

	Full sample at pre-treatment (*n* = 59)	Completed quantitative assessment at 10-month follow-up (*n* = 17)	Interviewed for qualitative analysis (*n* = 10)
Age (years): *M* (SD) Range: *Min-Max*	37.5 (11.4)	33.5 (9.3)	33.4 (9.1)
	20–69	20–49	20–49
Sex: *n* (% male)	34 (58%)	7 (41%)	4 (40%)
Highest Educational Level: *n* (%)			
Elementary school	2 (7%)	1 (6%)	1 (10%)
Upper secondary school	23 (39%)	7(41%)	3 (30%)
Vocational education	2 (7%)	1 (6%)	0
University (ongoing)	8 (14%)	3 (18%)	1 (10%)
University (completed)	19 (32%)	4 (24%)	4 (40%)
Other	5 (8%)	1 (6%)	1 (10%)
Occupation: *n* (%)			
Student	22 (37%)	10(59%)	5 (50%)
Employed	10 (17%)	2(12%)	2 (20%)
Unemployed	14 (24%)	2 (12%)	1 (10%)
Sick leave (> 3 months)	2 (3%)	1 (6%)	1 (10%)
Parental leave	1 (2%)	2 (12%)	1 (10%)
Other	10 (17%)	0	0
Prior psychological treatment: *n* (% yes)	9 (15%)	6 (36%)	4 (40%)
Taking medication: *n* (%)			
Yes	10 (17%)	4 (24%)	2 (20%)
No, but previously	6 (10%)	2 (12%)	1(10%)
No	43 (73%)	11 (65%)	7(70%)
Born in Sweden: *n* (%)			
Yes	0	0	0
No	59 (100%)	17 (100%)	10 (100%)
Immigration status: *n* (%)			
Immigrant	4 (7%)	2 (12%)	1 (10%)
Refugee	46 (78%)	13 (77%)	8 (80%)
Do not want to answer	9 (15%)	2 (12%)	1(10%)

### Intervention

The treatment was an 8 week long individually tailored ICBT treatment that had been culturally adapted for the target audience. Given the diversity present among Arabic-speaking re-settled persons, the focus of the adaptation process was to make the case examples, treatment material and Arabic translation accessible and recognizable for a wide range of participants. For more details on the cultural adaptation, see [28, 43]. The treatment was a guided treatment with the therapists consisting of two psychologists and two master’s degree-level psychologist students. The participants received weekly feedback on their homework assignments by way of nonsynchronous text messages sent through an in-built messaging system in the treatment platform. The treatment consisted of 9 treatment modules, with one introductory module, one maintenance module as well as seven modules targeting the following problem areas; anxiety, depression, sleep problems, stress, worry and rumination, traumatic memories and emotion regulation difficulties. Each module consisted of a brief description of the problem area followed by a CBT conceptualization of the psychological mechanisms that cause and maintain the problem. Each module also contained specific exercises informed by the CBT conceptualization, such as exposure exercises in the anxiety module and behavioral activation exercises in the depression module. Participants were assigned modules based on a combination of their symptom presentation and own stated preference. In general, a new module was administered once a participant had completed the previous module. However, the tailoring was also modified along the way based on the participants’ response to the treatment. On average, participants completed 2.23 modules with ﻿the anxiety module being the most frequently assigned module (67% of participants) followed by the emotion regulation module (50%), depression (40%), worry (33%), difficult memories (23%), insomnia (23%) and stress (20%) modules.

### Quantitative measures

All quantitative measures were filled out online on an encrypted platform, for details see [44]. For the original RCT, the PHQ-9 [41] which measures depressive symptoms, was used as primary outcome measure. Secondary outcome measure of anxiety (GAD-7) [42] perceived stress (Percieved Stress Scale-14) [45], sleep problems (Insomnia Severity Index) [46] post-traumatic stress (Impact of Events Scale-Revised) [47] and quality of life (Brunnsviken Brief Quality of Life scale) [48] were also used. In addition, the Alcohol Use Disorders Identifications Test (AUDIT) [49] was used to screen for alcohol misuse at pre-treatment.

### Data collection

All interviews were conducted over telephone by a female master-level psychologist student (FK). The interviewer had previously conducted clinical interviews with some of the participants to determine eligibility for the original RCT and had also acted as a therapist for some participants in the guided ICBT intervention. Participants were informed about the research procedure and that the purpose of the interview was to get a better understanding of their experiences of the ICBT program, in order to further improve the treatment. All interviews were conducted in Arabic, according to the preference of the participants, and lasted for approximately 20 min each, with a range of 13 to 23 min. All interviews were recorded and translated into Swedish, and then transcribed. The transcripts were not returned to participants for comments or correction due to data security reasons. The interviews centred around six main open-ended questions that were asked to the participants:

1. What was your overall experience of the treatment?
2. Which aspects of the treatment did you find meaningful?
3. Have you been able to make use of what you have learned during treatment?
4. Do you have suggestions for improvement of the treatment program?
5. Describe your view of mental health problems before treatment and after the treatment.
6. What is your view of seeking help for mental health problems in the future?

When needed, clarifying questions were used to deepen the understanding of the participants’ experiences. These questions were chosen in order to better understand both positive and negative aspects of participants’ experience of the treatment program, as well as to better understand their view of mental health problems and help-seeking, given the stigma surrounding mental health problems in Arabic culture [13, 14], and the potential for ICBT to act as a precursor for accessing other healthcare [33].

### Data analysis

The qualitative data was analysed using Thematic Analysis [50]. Thematic Analysis is a method for identifying and analysing recurring patterns or themes in data and is well-suited for the analysis of transcribed interview data. The analysis was made in an inductive, bottom-up, way, rather than a deductive, top-down, way, given the lack of previous research and theories in this area. This type of analysis is considered to be more reflective of the actual data, although it is also important to acknowledge that the analysis is influenced by the researchers own theoretical and epistemological orientation [50].

The analysis followed the six steps outlined by Braun and Clarke [50] which includes familiarising oneself with the data, initial coding of the data, searching for themes, reviewing of themes in relation to codes and the entire data set, defining and naming themes and the final write-up. The analysis was conducted by two of the authors (FK & SH), one of which had extensive knowledge of both the intervention itself as well as Arabic culture whereas the other author had extensive knowledge of cognitive behavioural therapy in general but not this specific intervention and was not familiar with Arabic culture in relation to mental health. The initial coding of the data into meaning units was conducted separately by the two authors and was subsequently combined into a joint coding document. Following this, both authors searched for themes independently which were then compared and merged into a single set of themes through consensus discussion. The remaining steps of the analysis were conducted jointly by the two authors. The analysis was reviewed and revised for the final write-up by a third author (TL), who also had extensive previous knowledge about the intervention. Other authors were involved in the write-up process and also took part in the initial formulation of the results section.

## Results

### Quantitative outcomes

In total, 17 of the 59 participants (29%) completed the 10-month assessment. Of these, 8 belonged to the initial treatment group and 9 to the wait-list control. However, as previously noted, by this time both groups had received the same treatment. The participants who did not complete the 10-month assessment were significantly more likely to be unemployed at pre-treatment, *χ*^*2*^(5, *N* = 59) = 16.25, *p* = .006, and also had a higher score on the AUDIT questionnaire *t* (57) = − 2.12, *p* = .038. For descriptive statistics of the outcome measures throughout the study and at the 10-month follow-up, see Table 2.

**Table 2:** Observed means, standard deviations and Ns for each measure for the two conditions over time

Measure and condition	Pre-treatment*			Post-treatment*		10-month Follow-up**			
	M	SD	N	M	SD	N	M	SD	N
**PHQ-9**									
*ICBT*	15.63	6.67	30	11.67	6.05	18	11.38	7.15	8
*wait-list*	17.79	5.29	29	17.33	5.29	18	9.33	7.5	9
**GAD-7**									
*ICBT*	13.07	4.73	30	9.11	5.46	18	8.9	6.85	8
*wait-list*	13.93	5.0	29	13.56	5.28	18	8.9	6.43	9
**PSS-14**									
*ICBT*	36.17	5.41	30	29.44	7.16	18	27.5	11.15	8
*wait-list*	38.21	6.18	29	36.78	6.84	18	29.78	9.68	9
**ISI**									
*ICBT*	15.53	6.07	30	11.72	6.69	18	15.13	8.36	8
*wait-list*	16.59	6.21	29	15.72	7.15	18	10.11	8.58	9
**IES-R**									
*ICBT*	52.23	17.58	30	45.61	19.23	18	51.0	25.17	8
*wait-list*	54.34	16.02	29	48.61	16.98	18	32.5	20.78	8
**BBQ**									
*ICBT*	38.77	17.91	30	50.22	18.58	18	45.6	23.3	8
*wait-list*	34.17	20.46	29	33.61	16.74	18	52.88	32.22	8

*Note.* PHQ-9 = Patient Health Questionnaire-9; GAD-7 = Generalized Anxiety Disorder-7; PSS-14 = Percieved Stress Scale-14; ISI = Insomnia Severity Index; IES-R Impact of Events Scale-Revised; BBQ = Brunnsviken Brief Quality of Life Scale

* Data previously presented in Lindegaard et al., (2020)

** At the 10-month follow-up, both groups had received the same treatment

### Thematic analysis

#### Overall results

The thematic analysis resulted in five main themes; 1) *The importance of being seen,* 2) *New ways of knowing and doing,* 3) *Treatment format not for everyone,* 4) *Changing attitudes towards mental health and help-seeking* and 5) *The healthcare system as a complex puzzle,* which are presented below. All quotations are available in Arabic in Additional file 1.

#### 1) The importance of being seen

One important aspect of the treatment experience as expressed by participants related to their experience of being able to establish a mutual trusting relationship with their therapist. Some participants expressed satisfaction with the therapist’s engagement and how this contributed to a feeling of being seen.

*“... I felt safe, that there was someone who understood … there was someone who cared. You feel that your condition is recognized.”* (P5)

In contrast, other participants expressed that they would have wanted more contact throughout the treatment, preferably via telephone or in a physical meeting, and felt limited by only having contact via email.

“[ … ] *I probably work best when I have direct contact. It was a little difficult during that period to concentrate. But if I had had [telephone] conversations with my therapist then I would probably have felt more seen and that there is some connection between us, that I am understood [ … ] which could perhaps make me follow the treatment a little better.*” (P4)

As shown in this quote, for some participants, an increased connection with the therapist and a sense of being seen and understood could have helped facilitate engagement in and adherence to the treatment. When this connection failed to establish, this led to less motivation to engage with the treatment material.

#### 2) New ways of knowing and doing

Some participants described having gained increased knowledge and understanding of mental health problems. This included a new understanding of the causes to the problems experienced by the participants as well as an increased knowledge of the psychological and physical symptoms of common mental disorders, and thus an ability to label one’s own experience.

*“One thing that I thought was good in the study was that you kind of understand how it comes about that you get sick, [ … ] how come you get like this and that certain things that happen in life can affect you [ … ] so I just thought these texts that you got about, for example, a certain diagnosis or so, contained a lot of information, [ … ] you learned certain things you knew nothing about. "*(P8)

In addition, participation also seemed to result in increased self-awareness, where engagement with the treatment program enabled participants to self-reflect in new ways.

*“The questions were made in a way that allowed me to go inside and search within me for answers. Some of these questions I had never been able to ask myself before, I feel like I did not have access to them, so through the questions and taking part of all the advice in the texts that you got through the treatment, this encouraged me to search within me for answers.”* (P2)

Finally, participants also described new ways of doing things in their everyday life which seemed to impact them in a positive way. This included changing their routines, changes in their thinking patterns as well as focusing their attention in a different way, as exemplified by the following participant.

*“[ … ] maybe that you change routines and such, that was what was most helpful to me, when you change the direction from inside, self-focus, to starting to focus more outwards. When I did so, and became more aware of how I think, and directed the thoughts outwards and also when I stop thinking negative thoughts, and more positive ones, [I feel] more positive force, or energy.”* (P5)

As mentioned by this participant, changes in thinking patterns and new ways of focusing attention resulted in a sense of having more energy and a more positive feeling state, which was also echoed by other participants. One participant also mentioned an increased sense of direction and purpose in life.

#### 3) Treatment format not for everyone

Another theme that emerged was the experience of the internet treatment format and how it either contributed to, or hindered, progress. Some participants expressed satisfaction with the treatment format, including the flexibility to engage with the treatment at a time of their liking, as well as how the treatment was easy to understand and follow along.

*"... I felt that all the steps were simple, that it was laid it out in a good way, [ … ] it was no problem for me to follow along."* (P3)

Participants also expressed appreciation for the contact with their therapist and for the fact that the treatment was delivered in Arabic, which according to one participant was preferable to face-to-face therapy with an interpreter. However, several difficulties and limitations with the treatment format were also encountered including technical problems logging in to the treatment platform as well as difficulties understanding the questionnaire items and the treatment texts.

*“[ … ] but sometimes there were difficult words and terms used in the text so that it required an effort to go through and understand.”* (P5)

In addition, some participants also reported difficulties in applying the material that they learned in real life. Some participants reported that it was hard to understand how to perform some of the homework assignments while others reported that following the steps outlined did not produce the desired results.

*"So I tried to follow those steps, but sometimes I followed the steps but I didn't get much results, and sometimes I gave up."* (P8)

This quote also gives an indication that an experience of lack of desired result led to decreased motivation for this participant. Other participants reported difficulties engaging with the written treatment material due to symptoms related to their mental health problems, such as difficulties concentrating or high anxiety.

*“[ … ] in my situation, I had difficulties focusing, sometimes I could not take in what I read.”* (P9)

Similarly, some participants also reported stressful circumstances in their life situation that were not fully considered in the treatment format, which led to difficulties in focusing on the treatment and which exacerbated their mental health problems.

*“... my big problem is not in the treatment itself, my big problem is my situation here at home, how I feel now, how I live and this whole process I have gone through, that is what makes me continue to not feel well”* (P3)

As exemplified by the above quotation, some participants demonstrated a tendency to either blame themselves or external circumstances for lack of effect of the treatment on their well-being, rather than the treatment itself not being sufficiently tailored to their individual needs.

#### 4) Changing attitudes towards mental health and help-seeking

Consistent with the previous literature, participants also reported experiencing stigma surrounding mental health problems, which for some seemed to constitute a barrier for treatment seeking.

*"This was my first time [seeking help], I know that it is very difficult as many in my community feel that this is something unknown or even have a fear of contacting a psychologist"* (P1)

However, participants also differed regarding to what extent they self-identified with this view, and some also emphasized that mental health problems are normal and not something to feel ashamed about. Seeking help via the internet was considered more accessible due to the increased anonymity that it provides.

*“You feel safe online and it's personal, that's what was good about the study, no one sees and no one hears [ … ] No one looks at the person negatively. Personally, I do not mind mental illness or that sometimes you need to seek help” (*P5)

Participants also noted a need for professional help when suffering from mental health problems and the importance of addressing these problems. They also mentioned an increased awareness that it was possible to receive help which, according to them, was a result of their participation in the study. This also included a more favorable attitude towards the healthcare system and towards seeking help for mental health problems.

*“... this whole experience gave me a new perspective when it comes to seeking help for mental health problems, I mean, I’ve started telling others around me [that] help was available. You do not have to keep feeling bad”* (P2)

As shown in this quote, this changed attitude towards help-seeking was not confined only to the participants themselves but also led to recommending other people to seek help for their mental health problems.

#### 5) The healthcare system as a complex puzzle

The last theme pertains to participants’ previous experiences of seeking help for mental health problems and the difficulties that they’ve encountered in this process. These difficulties include a lack of knowledge what help is available through the healthcare system and difficulties establishing contact with the healthcare system.

*“It is not so easy when you are newly arrived, not so easy when you do not know much about how the system works here.”* (P3)

*“[* … *] but at the same time it is difficult to get through, I had to seek help several times before I finally received it.”* (P9)

In addition, some participants also report difficulties being understood once contact is established, as exemplified by the following quote.

*“I think I have good knowledge of Swedish but when I seek healthcare or other help, I still think it has been very difficult when you cannot make yourself understood, even when I have an interpreter I feel that what I want to say is not understood, the interpreter does not convey what I want to convey.”* (P2)

In this example, even the use of an interpreter is not always sufficient to bridge the gap in understanding between participants and healthcare professionals, with resulting frustration and inability to receive the care that one needs. This difficulty also speaks to the complexity of conveying emotional experiences to healthcare professionals and the importance of the latter having an understanding of the patients’ cultural context in order to facilitate communication surrounding these complex issues.

## Discussion

The current study was, to our knowledge, the first study to investigate the experiences of participating in an ICBT program among Arabic-speaking immigrants and refugees in Sweden using Thematic Analysis. The analysis resulted in five main themes which were labeled as 1) *The importance of being seen,* 2) *New ways of knowing and doing,* 3) *Treatment format not for everyone,* 4) *Changing attitudes towards mental health and help-seeking* and 5) *The healthcare system as a complex puzzle.*

Regarding the first theme related to the importance of being seen and understood by one’s therapist, some participants reported being able to do so while others felt they would have needed more contact, preferably via phone or in a physical meeting. This finding mirrors results from previous qualitative studies on ICBT where some participants experience the therapist contact as satisfactory while others report a wish for more human contact and interaction throughout the treatment [40]. These results also relate to findings from a previous study on ICBT for post-traumatic stress among Arab war-traumatized patients where higher working alliance predicted better treatment outcome [51], as well as with meta-analytic evidence showing a similar strength of association between treatment alliance and outcome as in face-to-face therapy [52]. Whether or not this alliance is especially important for immigrant and refugee populations is, however, an open question and more research is needed to determine this. In addition, it is also important to note that the concept of working alliance goes beyond a feeling of being seen and understood by one’s therapist, and also includes factors such as agreeing on common goals and the tasks that make up therapy [53], which were not captured by this theme.

In the second theme, participants described acquisition of new ways of understanding themselves and their symptoms as well as new ways of thinking, doing and focusing their attention. This in turn seemed to lead to more positive feeling states as well as increased energy and purpose. This theme seem to reflect the overall positive outcomes obtained in the original trial [28] and indicates that at least some participants were able to translate the treatment material and homework exercises into their everyday lives in ways that were meaningful to them, similarly to results obtained in previous qualitative ICBT studies [31, 33, 35, 36, 39]. Interestingly, while some participants described the treatment as helping them find new answers *within* themselves, others described a decreased self-focus which was related to a feeling of having more energy. Though seemingly paradoxical, these statements might reflect complementary treatment processes, and underscores the fact that self-reflective cognitive processes can be helpful under some circumstances and unhelpful under other [54].

However, as described in the third theme, participants also differed in their perception of the usefulness of the treatment format. In line with findings in previous qualitative ICBT studies [40], some participants expressed appreciation for the flexibility inherent in the format and found the treatment easy to follow along. In contrast, other participants instead found parts the text material hard to understand and also experienced difficulties applying the information in real life, which sometimes seemed to lead to a loss of motivation. A similar theme was found by Rozental et al. [37] related to implementation of tasks assigned during treatment where some patients reported feeling like failures after unsuccessful implementation attempts and questioned whether they would ever recover from their condition or benefit from the treatment. Similar to participants in the present study, Rozental et al. [37] also highlighted that many participants seemed to attribute these difficulties to personal characteristics rather than shortcomings in the treatment program. These findings point to the importance of close monitoring of participants progress throughout the treatment program and the importance of addressing and examining difficulties with application of treatment principles together with the participants in a non-judgmental, explorative way. Another important implication has to do with the marketing of ICBT programs. Given that previous research has shown that a substantial proportion of participants do not experience significant treatment gains, it might be important to inform participants about this before commencing treatment so that they can have a realistic expectation of the treatment process and that the treatment is not likely to help everyone.

Another aspect of the treatment format explored under this theme is how some participants described how stressful life circumstances and symptoms such as high anxiety and difficulties concentrating interfered with their ability to engage with the treatment program. Some of these difficulties could be related to the various post-migrations stressors that migrants and refugees often experience, which have been linked to exacerbated symptoms of psychological disorders [6], and also associated with poorer treatment completion and lower treatment effects among resettled refugees [55]. These factors might have to be further considered and directly addressed in the treatment to make it more relevant and easier to apply for participants who experience a high degree of post-migration stressors.

Further, the fourth theme related to participants’ experience of mental health stigma and their view on seeking professional help. Consistent with previous research [10, 13, 14], many participants described experiencing some level of stigma surrounding mental health problems although participants differed to what extent they themselves had internalized with this stigma, so called self-stigma, which has been linked in previous research to reduced help-seeking [14]. For some, this stigma had previously constituted a barrier to seeking help. For this reason, the anonymity provided by the internet format was seen as helpful in this regard. Several participants also expressed that participation in the study gave them a more favorable view and understanding of mental health problems and a more positive attitude towards seeking help for psychological problems. As mentioned above, a similar theme was described by Darvell et al. [33] where participants described participation in the ICBT program as facilitating other treatment seeking. Notably, none of the participants mentioned traditional ways of explaining mental health problems which often include religious and supernatural explanatory models [13], instead all participants seemed to favor bio-psycho-social models for understanding their mental health symptoms. However, this might not necessarily reflect the views held in these communities at large, rather, it might be an expression of self-selection bias where people who adhere to religious or supernatural explanations of mental health symptoms choose not to participate in ICBT programs.

Finally, the fifth theme related to participants’ past experience of seeking help for their mental health problems and the difficulties that they experienced with navigating the healthcare system and being understood by professionals. Again, this finding is in line with previous research where lack of understanding of the healthcare system is often described as a barrier to accessing healthcare among immigrants and refugees [15, 56].

### Limitations

With regards to the limitations of the present study, as with all qualitative research it is important to note that the possibility to directly generalize the findings from the present study to other populations is limited [57], and is usually not the purpose of the research. However, the themes found within the present study give insight into possible ways of experiencing a culturally adapted ICBT treatment which can be used to further refine and develop these treatments. These developments can, in turn, be further studied using a combination of qualitative and quantitative methods.

A second limitation concerns the sample in the present study given that only a minority of the participants in the original trial completed the 10-month follow-up assessment and thus could be included in this study. The fact that non-completers had higher levels of unemployment and alcohol use at pre-treatment compared to completers indicates that the sample interviewed does not fully capture the variation present in the treatment group as a whole. Related to this, 4 of the 10 participants in the interviewed sample had undergone previous therapy compared to 15% in the full sample, which might have influenced both their adherence to the intervention as well as the efficacy of the intervention itself. Moreover, it is possible that the 10 participants who chose to participate in the qualitative interviews overall had more positive experiences of the treatment which in turn made them more inclined to participate in the interviews.

A third and related limitation concerns the high attrition rate which precluded the possibility of conducting a quantitative follow-up to complement the qualitative interviews. This makes it difficult to assess the long-term effects of the treatment.

Fourth, it is possible and even likely that the previous knowledge of the authors regarding ICBT and mental health challenges facing Arabic-speaking immigrants and refugees influenced the results in some way. However, this does not necessarily constitute a problem since researcher subjectivity can be seen as a resource rather than a problem in Thematic Analysis [58].

Finally, it is important to note that some of the participants in the present study had been in the wait-list control group and thus had received treatment at the end of the study phase. This meant that the semi-structured interviews were conducted at 8 months post-treatment for these participants. However, it is unlikely that this affected the results of the present analysis in any systematic way.

## Conclusion

The present study explored experiences of participants in a culturally adapted ICBT study for symptoms of depression and anxiety. While the results are largely in line with previous qualitative studies on ICBT participants, they also highlight some of the challenges regarding ICBT treatment for this specific population, such as the prevalence of mental health stigma and refugee-specific stressors that can hinder engagement with treatment. Future research should investigate if a more explicit acknowledgement and focus on the specific stressors and barriers to engagement faced by refugees can help increase engagement with the treatment, as well as increase the likelihood of successful transfer of treatment principles into the daily lives of participants. The finding that ICBT can act as a precursor to other treatment seeking and reduce mental health stigma could also be investigated more systematically in quantitative studies.

## Supplementary Information

**Additional file 1.**

## Data Availability

The quotations in the present study are based on transcribed interviews which contain sensitive information, the data can therefore not be made publicly available.

## Abbreviations

ICBT: Internet-based cognitive behavioural therapy
RCT: Randomized controlled trial
DHI: Digital health intervention
PHQ-9: Patient health questionnaire 9
GAD-7: Generalized anxiety disorder questionnaire 7
AUDIT: Alcohol Use Disorders Identifications Test
