# Supplementary material for: Experiences of internet-based cognitive behavioural therapy for depression and anxiety among Arabic-speaking individuals in Sweden: a qualitative study
Source: BMC Psychiatry. 2021 Jun 3;21:288. doi: 10.1186/s12888-021-03297-w (PMC8173836; doi:10.1186/s12888-021-03297-w)
Supplement: Supplementary file 1 — Additional file 1. [file 12888_2021_3297_MOESM1_ESM.docx]

| Additional file 1 | | |
| --- | --- | --- |
| Quotations in English and Arabic | | |
| Theme | Quotation in English | Quotation in Arabic |
| The importance of being seen | *“Even though it was just an interview, I felt that… I was really listened to and that the therapist made an effort to understand my story and how I felt then”* | "على الرغم من أنها كانت مجرد مقابلة، شعرت أن ... تم الاستماع لي وأن المعالج بذل جهدًا لفهم قصتي وشعوري" |
| The importance of being seen | *“... I felt safe, that there was someone who understood…there was someone who cared. You feel that your condition is recognized.”* | "شعرت بالأمان، وأن هناك شخصًا ما يفهمني ... كان هناك من يهتم لي. تشعر أن حالتك مألوفة" |
| The importance of being seen | *I probably work best when I have direct contact. It was a little difficult during that period to concentrate. But if I had had [telephone] conversations with my therapist then I would probably have felt more seen and that there is some connection between us, that I am understood […] which could perhaps make me follow the treatment a little better.*” | "اعتقد ان تجاوبي سيكون أفضل عندما يكون الاتصال مباشر. كان التركيز علي صعباً بعض الشيء في تلك الفترة. ولكن باعتقادي لو أجريت محادثات [هاتفية] مع معالجتي، لشعرت وكانه يمكن مشاهدتي وأن هناك بعض الصلة بيننا وانه ممكن فهمي [...] لربما تمكنت من متابعة العلاج بشكل أفضل" |
| New ways of knowing and doing. | *“One thing that I thought was good in the study was that you kind of understand how it comes about that you get sick, […] how come you get like this and that certain things that happen in life can affect you […] so I just thought these texts that you got about, for example, a certain diagnosis or so, contained a lot of information, […] you learned certain things you knew nothing about. "* | "باعتقادي انه واحده من الاشياء الجيدة في الداراسة هي فهم الامور التي ادت الى مرضي، [...] كيف تحدث هذه الامور وكيف تؤثر بعض هذه الامور على مجرى حياتك [...] فان هذه النصوص، على سبيل المثال، حول التشخيص أو نحو ذلك، تحتوي على الكثير من المعلومات، [...] وعلمتني أشياء جديدة لم اعرفها من قبل" |
| New ways of knowing and doing. | *“The questions were made in a way that allowed me to go inside and search within me for answers. Some of these questions I had never been able to ask myself before, I feel like I did not have access to them, so through the questions and taking part of all the advice in the texts that you got through the treatment, this encouraged me to search within me for answers.”* | "تم طرح الأسئلة بطريقة سمحت لي بالدخول والبحث في داخلي عن إجابات. لم أتمكن من طرح بعض هذه الأسئلة على نفسي من قبل، شعرت بعدم قدرتي على الوصول إليها ، لذلك من خلال الأسئلة والنصائح الواردة في النصوص التي تلقيتها من خلال العلاج، شجعني ذلك في البحث عن الاجابات في داخلي." |
| New ways of knowing and doing. | *“[…] maybe that you change routines and such, that was what was most helpful to me, when you change the direction from inside, self-focus, to starting to focus more outwards. When I did so, and became more aware of how I think, and directed the thoughts outwards and also when I stop thinking negative thoughts, and more positive ones, [I feel] more positive force, or energy.”* | "[...] عندما غيرت نظام يومي المعتاد وما إلى ذلك، كان هذا الشيء الأكثر فائدة بالنسبة لي، مكني ذلك من تغيرتوجهي الداخلي، والتركيز على الذات والخارج. عندما فعلت ذلك، أصبحت مدركاً اكثر حول كيفية تفكيري، وجهت الأفكار للخارج وتوقفت عن التفكير بشكل سلبي والتفكير بطريقة ايجابية اكثر، [أشعر] بقوة أو طاقة إيجابية اكثر" |
| Treatment format not for everyone | *"... I felt that all the steps were simple, that it was laid it out in a good way, […] it was no problem for me to follow along."* | "... شعرت أن جميع الخطوات كانت بسيطة، وأنه تم وضعها بطريقة جيدة، [...] لم يكن هناك مشكلة بالنسبة لي في المتابعة." |
| Treatment format not for everyone | *“[…] but sometimes there were difficult words and terms used in the text so that it required an effort to go through and understand.”* | "[...] ولكن في بعض الأحيان كانت هناك كلمات ومصطلحات صعبة مستخدمة في النص بحيث تتطلبت جهدًا لتصفحها وفهمها." |
| Treatment format not for everyone | *"So I tried to follow those steps, but sometimes I followed the steps but I didn't get much results, and sometimes I gave up."* | "لقد حاولت اتباع هذه الخطوات، ولكن في بعض الأحيان لم أحصل على الكثير من النتائج، وأحيانًا استسلمت." |
| Treatment format not for everyone | *“[…] in my situation, I had difficulties focusing, sometimes I could not take in what I read.”* | "[...] في حالتي، واجهت صعوبات في التركيز، وأحيانًا لم أستطع استيعاب ما أقرأه." |
| Treatment format not for everyone | *“... my big problem is not in the treatment itself, my big problem is my situation here at home, how I feel now, how I live and this whole process I have gone through, that is what makes me continue to not feel well”* | "... مشكلتي ليست في العلاج نفسه، مشكلتي الكبيرة هي ما اعاني منه هنا في المنزل، كيف أشعر الآن، والتجربة التي مررت بها، هذا ما يجعلني بعدم الشعور بحال جيدة" |
| Changing attitudes towards mental health and help-seeking. | *"This was my first time [seeking help], I know that it is very difficult as many in my community feel that this is something unknown or even have a fear of contacting a psychologist"* | "كانت هذه أول مرة [طلب المساعدة] ، أعلم أن الأمر صعب للغاية حيث يشعر الكثير في مجتمعي أن هذا شيء خارج عن المألوف أو حتى يخافون من التواصل مع معالج نفسي" |
| Changing attitudes towards mental health and help-seeking. | *“You feel safe online and it's personal, that's what was good about the study, no one sees and no one hears […] No one looks at the person negatively. Personally, I do not mind mental illness or that sometimes you need to seek help”* | "تشعر بالأمان والخصوصية عبر الإنترنت، وهذا ما كان جيدًا في الدراسة، وليس هنالك احد يراك اويسمعك [...] ولا تشعر بان هنالك احد ينظراليك بصورة سلبية. أنا شخصياً لا أمانع المرض النفسي أو أنك في بعض الأحيان تحتاج إلى طلب المساعدة " |
| Changing attitudes towards mental health and help-seeking. | *“... this whole experience gave me a new perspective when it comes to seeking help for mental illness, I mean, I’ve started telling others around me [that] help was available. You do not have to keep feeling bad”* | "... اكسبتني هذه التجربة نواح جديدة بطلب المساعدة لمرض نفسي، واخبرت الآخرين من حولي [أن] المساعدة كانت متاحة. ليس من الضروري ان تستمر في الشعور بالسوء " |
| The healthcare system as a complex puzzle. | *"It is not so easy when you are newly arrived, not so easy when you do not know much about how the system works here."* | "ليس الأمر سهلا انك وافد جديد، وليس بهذه السهولة عندما لا تعرف الكثير عن كيفية عمل النظام هنا." |
| The healthcare system as a complex puzzle. | *“[*…*] but at the same time it is difficult to get through, I had to seek help several times before I finally received it.”* | "[...] ولكن في نفس الوقت يصعب الاستمرار، اضطررت إلى طلب المساعدة عدة مرات قبل أن أحصل عليها." |
| The healthcare system as a complex puzzle. | *“I think I have good knowledge of Swedish but when I seek healthcare or other help, I still think it has been very difficult when you cannot make yourself understood, even when I have an interpreter I feel that what I want to say is not understood, the interpreter does not convey what I want to convey.”* | "أعتقد أن لدي معرفة جيدة باللغة السويدية ولكن عندما أطلب الرعاية الصحية أو أي مساعدة أخرى، ما زلت أعتقد أنه امر صعباً للغاية لا يمكنني التعبيرعن نفسي، حتى عندما يكون هنالك مترجم موجود، أشعر أن ما أريد قوله غير مفهوم وان المترجم لا ينقل ما أريد أن أنقله ". |
